# MUC1* Mediates the Growth of Human Pluripotent Stem Cells

**DOI:** 10.1371/journal.pone.0003312

**Published:** 2008-10-03

**Authors:** Sherry T. Hikita, Kenneth S. Kosik, Dennis O. Clegg, Cynthia Bamdad

**Affiliations:** 1 Center for Stem Cell Biology and Engineering, University of California Santa Barbara, Santa Barbara, California, United States of America; 2 Department of Molecular, Cellular and Developmental Biology, University of California Santa Barbara, Santa Barbara, California, United States of America; 3 Neuroscience Research Institute, University of California Santa Barbara, Santa Barbara, California, United States of America; 4 Minerva Biotechnologies, Waltham, Massachusetts, United States of America; Dresden University of Technology, Germany

## Abstract

The MUC1 protein is aberrantly expressed on an estimated 75% of all human solid tumor cancers. We recently reported that a transmembrane cleavage product, MUC1*, is the predominant form of the protein on cancer cells [1]. Further, our evidence indicated that MUC1* functions as a growth factor receptor on tumor cells, while the full-length protein appeared to have no growth promoting activity. Here, we report that MUC1* acts as a growth factor receptor on undifferentiated human embryonic stem cells (hESCs). Cleavage of the full-length ectodomain to form MUC1*, a membrane receptor, appears to make binding to its ligand, NM23, possible. Unexpectedly, we found that newly differentiated cells no longer express the cleaved form, MUC1*, or its ligand, NM23. Newly differentiated stem cells exclusively present full-length MUC1. Antibody-induced dimerization of the MUC1* receptor on hESCs stimulated cell growth to a far greater degree than currently used methods that require the addition of exogenous basic fibroblast growth factor (bFGF) as well as factors secreted by fibroblast “feeder cells”. Further, MUC1* mediated growth was shown to be independent of growth stimulated by bFGF or the milieu of factors secreted by feeder cells. Stimulating the MUC1* receptor with either the cognate antibody or its ligand NM23 enabled hESC growth in a feeder cell-free system and produced pluripotent colonies that resisted spontaneous differentiation. These findings suggest that this primal growth mechanism could be utilized to propagate large numbers of pluripotent stem cells for therapeutic interventions.

## Introduction

Stem cells are classified as totipotent, pluripotent or multipotent. A totipotent stem cell, such as a fertilized egg, is capable of developing into a complete organism. Pluripotent stem cells, exemplified by undifferentiated embryonic cells, are able to develop into any cell or tissue type. Multipotent stem cells, found for example in bone marrow, are able to develop into a limited subset of cell types. Pluripotent stem cells hold the greatest promise for therapeutic use because they possess the ability to become virtually any cell type in the human body. In principal, pluripotent stem cells could be used to replace damaged tissues in organs that have traditionally been thought not to have a significant potential for functional self-repair such as heart muscle, spinal cord, brain tissue and kidney [2]–[6]. However, to implement these therapies, one must have the ability to produce a replenishable supply of pluripotent stem cells, on a large scale, that can then be induced to differentiate into the desired cell types. Certain technical hurdles must be overcome before clinical therapies using pluripotent stem cells can become a reality.

First, improved methods for propagating pluripotent stem cells and ensuring their pluripotency must be developed. Currently, it is not possible to culture embryonic stem cells (ESCs) without initiating some degree of spontaneous differentiation. Growing ESCs under optimized conditions yields only about 65–75% undifferentiated, pluripotent stem cells. The remainder spontaneously differentiate. This is a problem because the cells that have initiated differentiation appear to secrete factors that encourage neighboring cells to also differentiate. To maintain a useful supply of pluripotent stem cells, the undifferentiated colonies, or portions of those colonies, must be manually dissected away from those that have begun to differentiate, then re-plated for further growth. This process is labor intensive and inaccurate because it depends upon the technician's visual assessment of cell and colony morphology in the determination of which colonies remain undifferentiated. An additional problem is that there is an upper limit of about 100 generations that embryonic stem cells can be passaged before they lose pluripotency. Higher passage numbers often correlate with increased risk of abnormal karyotypes or genetic drift, wherein abnormal cells with a selective growth advantage overtake and suppress the pluripotent population [7].

The state of the art for culturing hESCs requires the addition of a milieu of poorly understood factors from fibroblast “feeder cells”. Some of these factors appear to be necessary to maintain the undifferentiated state, while others likely trigger differentiation. Factors secreted from fibroblasts are supplied either by growing the hESCs over a layer of fibroblast feeder cells [8] or by growing the stem cells over matrigel-coated surfaces and feeding with growth media that has been supplemented with conditioned media from fibroblasts [9]. Basic fibroblast growth factor (bFGF) has been identified as a mitogenic factor that helps maintain cultures in the undifferentiated state and is added to stem cell growth media for optimal yield of undifferentiated stem cells [10].

There is also the need for improved methods for identifying and isolating pluripotent stem cells from a mixed pool of undifferentiated and differentiated cells. It is evident that local environment plays a critical role in the process of stem cell differentiation. Pluripotent stem cells can be influenced to differentiate into particular cell types when grown over more mature cells [11]. It may be that transplanted pluripotent stem cells differentiate into the cell type of the local environment in response to factors secreted by those cells/tissues. One can imagine that the contamination of pluripotent stem cells with even a small number of cells that have already committed to a particular differentiation pathway would be sufficient to confound the ordered differentiation process in a way that could have disastrous outcomes. Whether stem cells are transplanted as pluripotent or at an intermediate stage of tissue type differentiation, successful stem cell treatments may benefit from pure populations of stem cells and a detailed understanding of the molecular triggers that initiate the various steps along the path from undifferentiated to mature cell. Reliable, high throughput methods for rapidly identifying and isolating pluripotent stem cells, in a manner that preserves cell viability, have not yet been developed.

Therefore, what are needed are methods to identify and isolate pluripotent stem cells, to propagate pure populations in the undifferentiated state and to understand the molecular mechanisms that maintain pluripotency as well as those that trigger differentiation. To that end, we focused upon mechanisms that drive malignant growth related to so-called tumor stem cells. We focused on a cell surface protein that appears to mediate the growth of a large class of cancer cells and asked if it also mediates the growth of stem cells. This protein, MUC1, is a transmembrane protein that is expressed on normal epithelia that line the respiratory, reproductive and gastrointestinal tracts. On healthy tissue, MUC1 is clustered at the apical border. But, on cancerous tissues MUC1 is over-expressed and uniformly distributed over the entire cell surface [12], [13]. An estimated 75% of all human solid tumor cancers aberrantly express the MUC1 protein [14]. The role of MUC1 in the healthy state has not been studied as extensively as its role in cancer, where there is significant evidence that it promotes tumor cell growth and survival [15]–[18].

In a recent article, we showed that a membrane-anchored MUC1 cleavage product, MUC1*, that retains only 45 amino acids of the original extracellular domain, is the predominant form of the protein on human cancerous tissues; the bulk of the extracellular domain is cleaved and shed from the tissue surface [1]. We further demonstrated that MUC1* has growth factor receptor-like activity wherein ligand-induced dimerization of the short extracellular domain activates the MAP kinase signaling pathway and stimulates cell growth. Blocking the ligand binding site of the extracellular domain inhibited cell growth in a dose-dependent manner. The purpose of the present studies was to investigate the possible roles of the two forms of the MUC1 protein, MUC1* and full-length MUC1, in the growth and differentiation of human embryonic stem cells.

## Results

**Figure 1:**
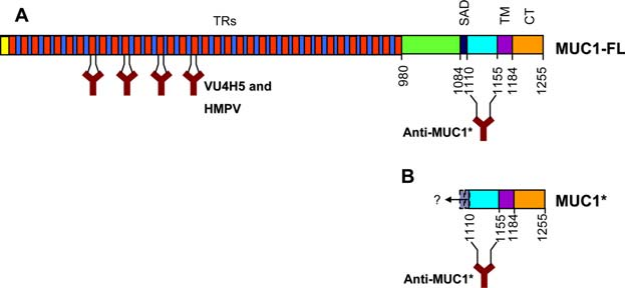
Schematic and antibody recognition of full-length MUC1 versus the membrane-bound cleavage product MUC1*. A. Full-length MUC1 protein (MUC1-FL) is comprised of a cytoplasmic tail (CT), a transmembrane domain (TM), a self-aggregation domain (SAD), and hundreds of tandem repeats (TRs). B. Cleavage product, MUC1*, consists of the cytoplasmic tail, transmembrane domain, and at least 45 amino acids of the extracellular domain (ECD). Although the exact site(s) of cleavage remain somewhat uncertain, to our knowledge, no cleavage sites have been reported that leave less than a 45 amino acid ECD. Binding sites for antibodies VU4H5 and Anti-MUC1* are marked.

We used three antibodies to probe the expression of MUC1 on hESCs: two that recognize the full-length protein (MUC1-FL) and one that recognizes the cleavage product, MUC1*. Both VU4H5 and HMPV are commercially available antibodies that bind to epitopes in the tandem repeats of the full-length protein (Fig. 1A). VU4H5 preferentially binds to underglycosylated MUC1, while HMPV recognizes full-length MUC1 in a glycosylation-independent manner and can bind to the fully glycosylated protein. Anti-MUC1* is a rabbit polyclonal antibody that was raised against a synthetic peptide that corresponds to the first forty-five (45) membrane-proximal amino acids of the extracellular domain, which comprises most if not all of the extracellular domain of the cleavage product, MUC1* (Fig. 1B). As we previously reported [1], although the epitope for Anti-MUC1* is present in the full-length protein, Anti-MUC1* does not bind to MUC1-FL when analyzed by Western blot or immunocytochemistry. Immunoprecipitation experiments show that Anti-MUC1* reacts very weakly with MUC1-FL. One possible explanation is that full-length MUC1 contains a self-aggregation domain that likely contributes to the protein's characteristic clustering and could sterically hinder the binding of ligands to the adjacent region which is the Anti-MUC1* epitope. Cleavage of MUC1 on cancer cells releases the bulk of the extracellular domain, including most if not all of the self-aggregation domain. It is not known whether or not Anti-MUC1* binds to alternative splice isoforms, such as MUC1/X, MUC1/Y or MUC1/Z [19], [20], which, like MUC1*, contain the Anti-MUC1* epitope and lack the tandem repeat region; one way that they differ from MUC1* is that their extracellular domains are more than one hundred (100) amino acids longer and contain the self-aggregation domain. Although MUC1/Y is not cleaved, it has been reported that MUC1/X/Z can be cleaved to yield a membrane-attached fragment that is essentially indistinguishable from the MUC1-FL cleavage product [21], and thus would likely be recognized by Anti-MUC1*.

### MUC1 Expression on Human Embryonic Stem Cells

Immunofluorescent cytochemistry experiments were performed on H9 and H14 human embryonic stem cells (hESCs). The stem cells were probed with Anti-MUC1*, VU4H5 and HMPV as well as with antibodies against a panel of known markers of the undifferentiated state [22], [23]. Both undifferentiated and differentiated hESCs were analyzed. Undifferentiated hESCs were obtained by culturing them under conditions that have been shown to support the undifferentiated state, followed by manual dissection of colonies that morphologically appeared to be undifferentiated, and plating onto chamber slides for further growth and antibody staining. At this final stage, cells were deemed to be undifferentiated if they stained positive for OCT4 [24]. hESCs were induced to differentiate by withholding exogenous bFGF for 14 days or longer [25]. Differentiation was confirmed, by visual inspection, testing negative for the presence of OCT4, and testing positive for the presence of the three germline markers: alpha fetoprotein for endoderm [26], smooth muscle actin for mesoderm [27], and nestin for ectoderm [28]. Stem cells used in these experiments were shown to be of normal karyotype (Fig. S1).

**Figure 2:**
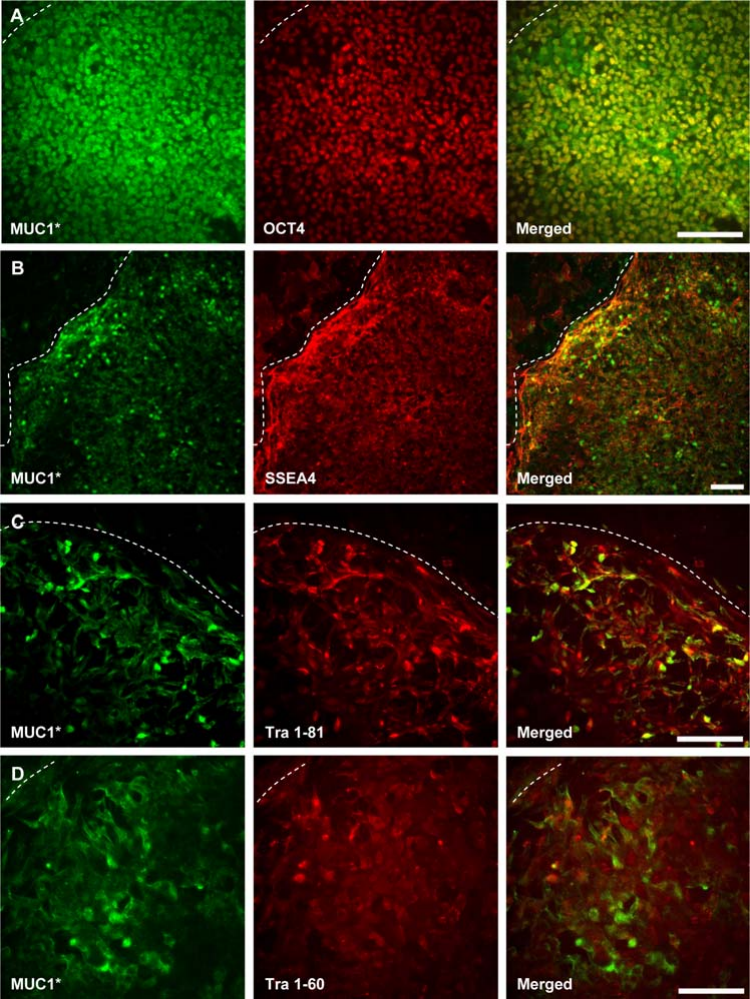
MUC1* co-localizes with OCT4 and other markers of pluripotency on undifferentiated human embryonic stem cells (hESCs). Undifferentiated H9 hESCs were cultured according to standard methods on chamber slides then double stained with Anti-MUC1* and antibodies that recognize known markers of pluripotency: A. MUC1* is co-expressed with OCT4 on undifferentiated hESCs. B. MUC1* and SSEA4 co-localize to a great extent on undifferentiated hESC colonies. C. MUC1* and Tra 1–81 partially co-localize on undifferentiated hESC colonies. D. MUC1* and Tra 1–60 are co-expressed on hESCs. Dotted lines indicate the border of the undifferentiated colony. Scale bars = 100 µm.

Immunocytochemistry experiments showed that MUC1* is highly expressed on undifferentiated hESCs; double staining experiments with an antibody against OCT4 confirmed that the cells that expressed MUC1* were in fact undifferentiated stem cells (Fig. 2A). DAPI staining revealed that both MUC1* and OCT4 were expressed by essentially all the undifferentiated cells (data not shown). Figure 2B shows that MUC1* expression also co-localized with SSEA4, another marker for undifferentiated hESCs [29], although expression between these two did not always precisely co-localize. MUC1* co-localized to a similar extent on undifferentiated hESCs with Tra 1–81 and Tra 1–60 [30] which are also indicators of the undifferentiated state (Figs. 2 C,D). MUC1* appears to be surface-expressed as evidenced by antibody staining in the absence of added detergent (Figs. 2 B–D). Control experiments for all immunocytochemistry imaging are shown in Figure S6.

**Figure 3:**
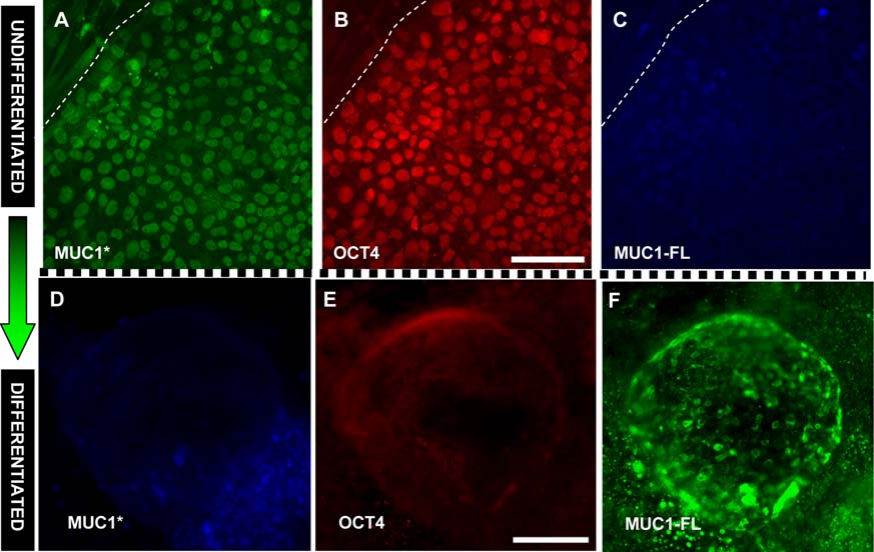
The cleaved form, MUC1*, is expressed on undifferentiated human embryonic stem cells while the full-length, uncleaved protein (MUC1-FL) is expressed on differentiated stem cells. A–C. Undifferentiated H9 hESC colonies were triple stained with antibodies against: A. MUC1*, B. OCT4, and C. MUC1-FL. The dotted line indicates the border of the undifferentiated colony. D–F. H9 hESC colonies were induced to differentiate by withholding bFGF for 14 days. Visual inspection indicated that the colonies had differentiated. The hESC colonies were triple stained with antibodies against: D. MUC1*, E. OCT4, and F. MUC1-FL. Scale bars = 100 µm.

Unexpectedly, we found that although the cleaved form, MUC1*, is highly expressed on undifferentiated hESCs, no full-length MUC1 was detectable. Undifferentiated stem cells stained positive for MUC1* and OCT4 but negative for full-length MUC1 (Fig. 3A–C). In stark contrast, newly differentiated embryonic stem cells exclusively expressed full-length MUC1 but not the cleaved form, MUC1*. Differentiating stem cells tested positive for full-length MUC1 and negative for both MUC1* and OCT4 (Fig. 3D–F). No full-length MUC1 was detectable on undifferentiated H9 or H14 hESCs, whether probed with the VU4H5 antibody or HMPV; after differentiation, both cell lines stained positive for MUC1-FL using either antibody (Fig. S2 A–H; Fig. S3 A–F). Both cell lines stained positive for the presence of MUC1* in the undifferentiated state, but not in the differentiated state (data not shown). We cannot rule out the possibility that Anti-MUC1* is also staining an alternative splice isoform such as MUC1/X, MUC1/Y or MUC1/Z. However, Western blot analysis of H9 hESCs revealed a 20 kD Anti-MUC1* reactive species that co-migrated with the MUC1 cleavage product from T47D breast cancer cells and with transfected MUC1*_1110_, that contains only forty-five (45) amino acids of the extracellular domain (Fig. S4). It follows that this low molecular weight species is the cleavage product of full-length MUC1, since it runs with an apparent molecular weight that is roughly half the apparent molecular weight of MUC1/Y and MUC1/Y is not cleaved.

**Figure 4:**
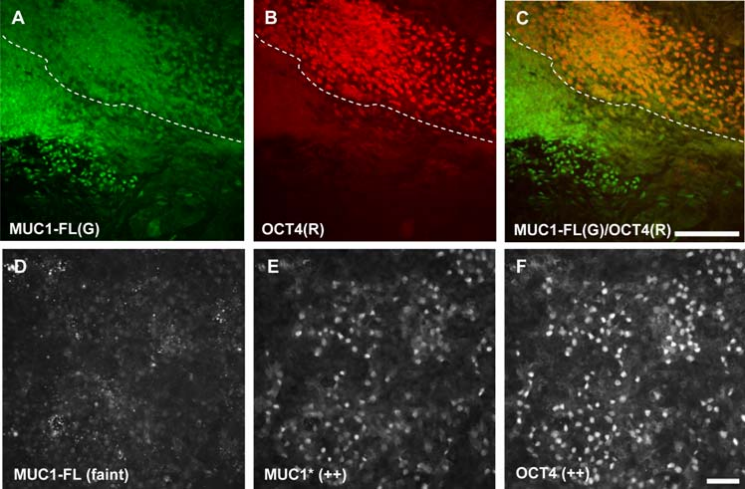
Transition zones between undifferentiated and differentiated hESCs simultaneously express full-length MUC1 and OCT4. hESCs grown without bFGF for 14 days appeared to be MUC1-FL positive and OCT4/MUC1* negative. However, rare transition regions were found that expressed all three proteins. A–C. The leading edge of an undifferentiated stem cell colony that has begun to differentiate stains positive for: A. MUC1-FL. B. OCT4. C. The merged image shows that MUC1-FL and OCT4 are expressed by the same cells. The border between undifferentiated and differentiated portions of the colony is marked by the dotted line. D–F. Another transition region simultaneously expressed: D. MUC1-FL. E. MUC1*. F. OCT4. However, tracking of individual cells indicates that MUC1* appears to faithfully co-localized with OCT4. Scale bars = 100 µm.

We further investigated the observed switch from MUC1* to MUC1-FL as stem cells enter the differentiation process. Closer inspection of many antibody-stained colonies revealed that there were rare transition regions that simultaneously expressed OCT4, the gold-standard marker for pluripotency, and full-length MUC1, which appears to be a marker for differentiation. Figure 4A–C shows that the edge of a colony that has begun to differentiate simultaneously expressed OCT4 and MUC1-FL. Other transition zones expressed MUC1-FL, MUC1* and OCT4 (Fig. 4D–I). It is notable that within these mixed populations, MUC1* appeared to faithfully co-localize with OCT4, while OCT4 sometimes co-localized with MUC1-FL.

### Expression of MUC1 Cleavage Enzymes on hESCs

**Figure 5:**
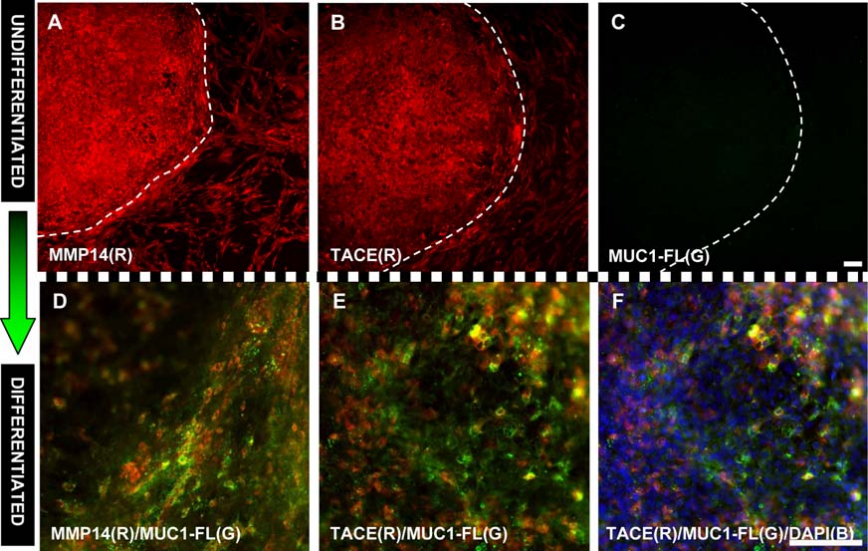
The expression of MUC1 cleavage enzymes, MMP14 and TACE, is high on undifferentiated hESCs but significantly lower on differentiated cells. A–C. Stem cells colonies that were deemed to be undifferentiated by visual inspection and by staining positive for the presence of OCT4 were stained with antibodies against MUC1-FL and the cleavage enzymes. A. Undifferentiated hESC colonies were stained with an antibody that recognizes MMP14. VU4H5, which recognizes MUC1-FL, did not stain this colony (data not shown). B. Undifferentiated hESC colonies were stained with an antibody that recognizes TACE. C. The same undifferentiated colony was also treated with VU4H5 (binds to MUC1-FL) but no immuno-reactivity was detected. D–F. Stem cells that were induced to differentiate by withholding bFGF for 14 days were stained with the same antibodies as in A–C. D. Newly differentiating stem cell colonies co-express MMP14 (red) and MUC-FL (green). E. Similarly, newly differentiating stem cell colonies co-express MUC1 cleavage enzyme TACE (red) and MUC1-FL (green). F. Triple staining of colonies with anti-TACE (red), VU4H5 (green) and DAPI (blue) showed that roughly 50% of the differentiating cells expressed the cleavage enzyme. An experiment using an antibody against MMP14 gave essentially the same result. Scale bars = 100 µm.

MMP14 (MT1-MMP) and TACE (ADAM 17) have been reported to be enzymes that cleave MUC1 on human uterine epithelial cells [31], [32]. If MMP-14 and TACE also cleave MUC1 on embryonic stem cells, then one might expect high expression levels on undifferentiated cells, where MUC1 is cleaved, and lower expression on differentiating cells where it is not. Immunofluorescent imaging revealed that both cleavage enzymes, MMP14 and TACE, are robustly expressed on undifferentiated stem cells that were completely devoid of full-length MUC1 (Fig. 5A–C). However, on newly differentiated stem cells, where MUC1-FL immunoreactivity was present, there was a marked decrease in MMP14 and TACE expression (Fig. 5 D–F). The merged image of a triple staining experiment, which also included DAPI staining, shows that approximately 50% of the cells present stained positive for the cleavage enzymes (Fig. 5F) compared to virtually 100% on undifferentiated colonies (data not shown). These findings support the idea that cleavage enzymes MMP14 and TACE cleave MUC1 on embryonic stem cells.

### MUC1* Ligand Expression on hESCs

**Figure 6:**
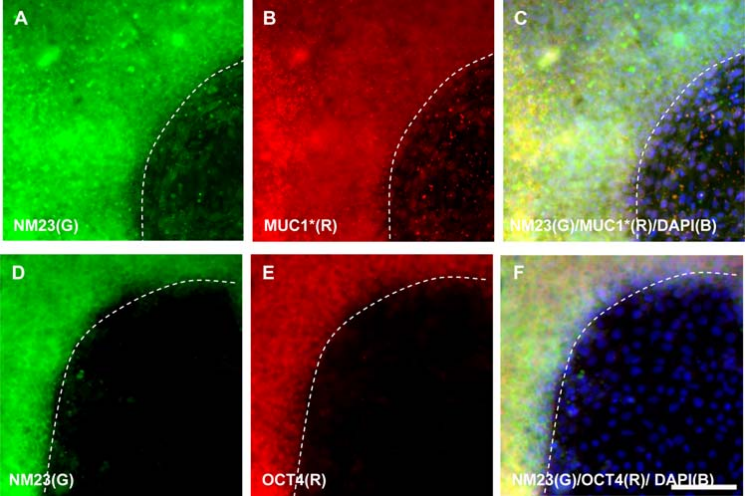
The MUC1* ligand, NM23, co-localizes with MUC1* and OCT4 on undifferentiated hESCs but immuno-reactivity of all three proteins is lost in the portion of the colony that has begun to differentiate. Undifferentiated H9 hESC colonies stained positive for NM23, MUC1* and OCT4. Newly differentiating colonies did not react with antibodies against any of the three proteins. Co-expression of NM23 with OCT4 and MUC1* is best seen in colonies that have begun to differentiate. The dotted line marks the border between undifferentiated and differentiated portions of the colonies. Triple staining experiments were performed using: A. anti-NM23 (green). B. Anti-MUC1* (red). C. anti-NM23 (green), anti-MUC1* (red) and DAPI (blue). A similar colony was stained with: D. anti-NM23 (green). E. anti-OCT4 (red). F. anti-NM23 (green), anti-OCT4 (red) and DAPI (blue). Scale bar = 100 µm.

NM23 is normally a cytoplasmic protein but is often secreted by tumor cells [33]. It can exist as a monomer, dimer, tetramer or hexamer, depending upon concentration [34]. NM23 has recently been identified as a ligand for MUC1* that stimulates the growth of tumor cells by dimerizing two MUC1* receptors [1]. We, therefore, looked for NM23 expression by hESCs. Figure 6 A–C depicts a triple staining experiment in which a hESC colony was stained with DAPI and antibodies against NM23 and MUC1*. The merged image (Fig. 6C) shows that expression of MUC1* and NM23 precisely co-localize, but are not expressed on newly differentiating cells at the edge of the colony. Another colony that had been stained with DAPI and antibodies against NM23 and OCT4 confirms that NM23-positive cells were in fact undifferentiated. Differentiated hESCs did not stain positive for the presence of NM23 (Fig. 6 D–F). These results are consistent with the idea that NM23 could also be a ligand of MUC1* on hESCs.

### MUC1*-Mediated Stem Cell Growth

We previously reported that [1], bivalent Anti-MUC1*, stimulated the growth of MUC1-positive tumor cells, whereas the monovalent Fab fragment of that same antibody potently inhibited growth. Experiments indicated that the bivalent antibody dimerized the MUC1* receptor, which activated the MAP kinase proliferation pathway, but the monovalent Fab blocked the interaction between MUC1* and its native ligand NM23. We were also able to demonstrate that NM23, and in particular the mutant that preferentially forms dimers [35], like the bivalent antibody, stimulated the growth of MUC1-positive tumor cells.

**Figure 7:**
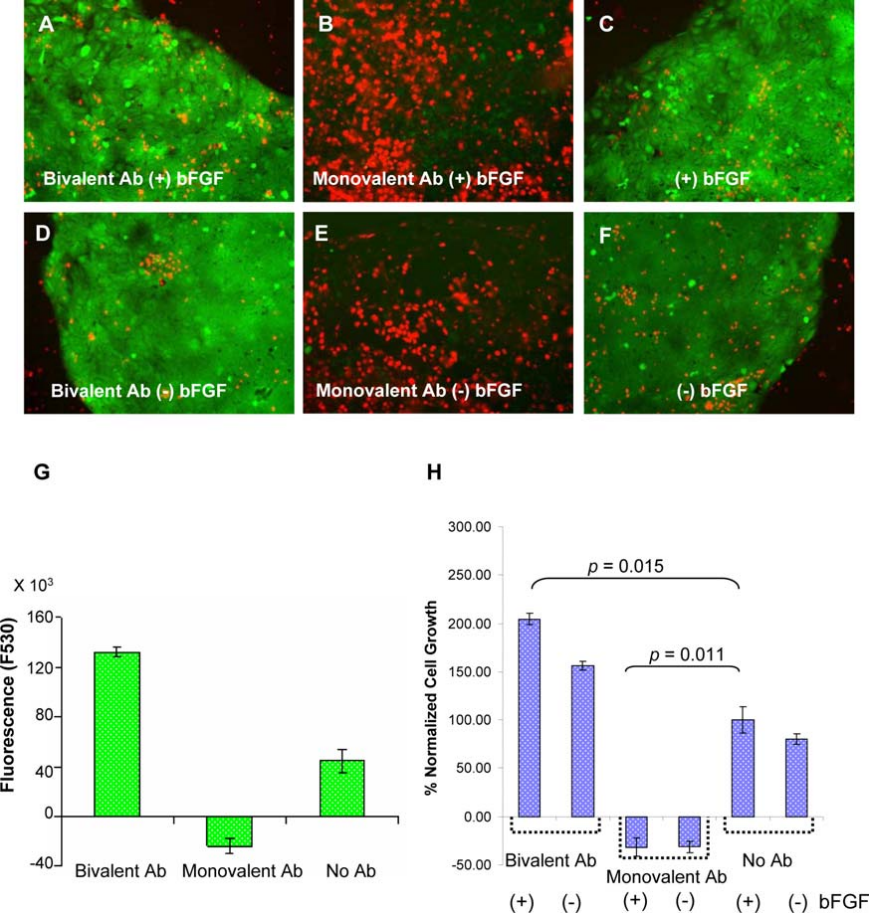
MUC1* mediates growth of pluripotent human embryonic stem cells. Undifferentiated H9 hESCs were grown for 41 hours in the presence of bivalent Anti-MUC1* (Bivalent Ab), which can dimerize the receptor, the monovalent Fab of Anti-MUC1* (Monovalent Ab), that blocks receptor dimerization, and/or basic fibroblast growth factor (bFGF). The results were quantified as follows. A–F. A live/dead (green/red) calcein assay was performed 41 hours post treatment. Photos record the results. A. Treatment with bivalent Anti-MUC1* (Bivalent Ab) and bFGF produced mostly viable cells (green) and very few dead cells (red). B. Treatment with the monovalent Fab of Anti-MUC1* (Monovalent Ab) and bFGF resulted in essentially total cell death within 12 hours. C. Treatment with bFGF alone produced mostly viable cells. D. Treatment with bivalent Anti-MUC1* alone produced more viable cells than with the addition of bFGF. E. Treatment with monovalent Anti-MUC1* killed essentially all cells. F. Treatment without antibodies or bFGF resulted in more dead cells and less viable cells. G. A bar graph shows that after 41 hours of treatment with Anti-MUC1* (Bivalent Antibody) there were more than 2-times the number of live cells than without the antibody (No Ab). Treatment with the monovalent Fab (Monovalent Ab) killed all the cells. Fluorescence of live cells in a calcein assay is plotted. Data are represented as mean fluorescence units±SEM. H. The percentage hESC growth is plotted for undifferentiated cells grown in the presence or absence of bFGF and with bivalent or monovalent Anti-MUC1* (Bivalent Ab, Monovalent Ab). Student's two-tailed test was used for statistical analysis. The graph shows that Anti-MUC1* with or without bFGF stimulated growth roughly twice as well as with bFGF.

We performed similar experiments to determine whether ligands of MUC1* could mediate the growth of pluripotent hESCs. Undifferentiated stem cells were grown on matrigel-coated wells and cultured according to current methods which included feeding with minimal stem cell media that had been supplemented with 30% conditioned media from Hs27 fibroblast feeder cells. Cells were treated with bivalent Anti-MUC1* or the monovalent Fab, in the presence or absence of exogenous bFGF. The addition of Anti-MUC1* to undifferentiated hESCs had a dramatic, stimulatory effect on cell growth. Treating hESCs with bivalent Anti-MUC1* for forty-one (41) hours, in the presence or absence of added bFGF, resulted in cells that were more viable and abundant than control cells that were cultured according to standard methods, which included adding bFGF. In stark contrast, the addition of the monovalent Fab fragment of Anti-MUC1* resulted in nearly total cell death within 12 hours of treatment (Fig. 7A–F). Presumably the monovalent Fab competed with MUC1*'s native ligand, NM23, for binding and blocked receptor dimerization. The growth effects of the MUC1*-targeting antibodies were quantified by measuring the fluorescence at 530 nm for live cells grown under each test condition. In the absence of added exogenous bFGF, the addition of bivalent Anti-MUC1* resulted in a greater than 2-fold enhancement of cell growth compared to the control (Fig. 7G). A plot of normalized cell growth with added bFGF defined as 100%, is shown in Figure 7H. Bivalent Anti-MUC1* greatly enhances the growth of undifferentiated stem cells and does not require the addition of exogenous bFGF. Notably, the addition of bFGF cannot rescue stem cells when treated with monovalent Anti-MUC1*. A similar experiment was performed in which the effects of bivalent Anti-MUC1*, its monovalent Fab, and a control Fab, on H9 and on H14 hESCs were measured (Fig. S5 A,B). The results were essentially the same as those depicted in Figure 7.

Since it appeared that MUC1*-mediated growth of hESCs was independent of the addition of basic fibroblast growth factor (bFGF), we investigated whether or not MUC1*-mediated growth required *any* fibroblast-derived factors. Specifically, we tested whether or not the addition of the MUC1* dimerizing antibody was sufficient to support the long-term growth of pluripotent hESCs in minimal media and in the absence of fibroblast feeder cells, their extracts, or purified bFGF. Undifferentiated H9 hESCs were plated at very low density onto matrigel and cultured for five weeks in either minimal stem cell media or minimal media supplemented with 30% conditioned media from Hs27 fibroblast feeder cells. The stem cells were treated every 48 hours with either: a) nothing; b) bFGF; c) bivalent Anti-MUC1*; or d) bFGF and bivalent Anti-MUC1*. Throughout the course of treatment, the plates were inspected for the appearance of new colonies. Their numbers and morphology were recorded and tabulated in Table 1.

**Table 1:** H9 hESCs cultured in minimal media for five weeks.

Growth Conditions	Time to First Visible Undifferentiated Colony				Morphology
	Week 1	Week 2	Week 3	Week 4	
30% Conditioned Media from Hs27 Fibroblast Feeder Cells					
nothing	-	-	-	-	3/4 wells fibroblast-like cells; 1/4 wells also had 1 small OCT4^−^ colony
bFGF 4 ng/ml	-	-	2/4 wells had colonies	growing	2/4 wells mixture of partially differentiated OCT4^+^/OCT4^−^ colonies; 2/4 wells fibroblast-like cells
anti-MUC1* 1 ug/ml	-	2/4 wells had colonies	fast growing	fast growing	More undifferentiated cells; 2/4 wells mixture of OCT4^+^/OCT4^−^ colonies+1 all OCT4^+^ colony; 2/4 wells fibroblast-like cells
bFGF 4 ng/ml & anti-MUC1* 1 ug/ml	-	-	-	-	4/4 wells OCT4^−^ fibroblast-like cells
Minimal Stem Cell Growth Media					
nothing	-	-	-	-	2/4 wells no live cells; 2/4 wells had OCT4^−^ fibroblast-like cells
bFGF 4 ng/ml	-	-	-	-	1/4 wells no live cells; 3/4 wells had OCT4^−^ fibroblast-like cells
anti-MUC1* 1 ug/ml	-	1/4 wells had colonies	fastest growing	fastest growing	largest colony; 1/4 wells 100% OCT4^+^ undifferentiated colony covered entire well – 1/4 wells OCT4^−^ fibroblast-like cells; 1/4 wells no live cells
bFGF 4 ng/ml & anti-MUC1* 1 ug/ml	-	-	-	-	2/4 wells no live cells; 2/4 wells OCT4^−^ fibroblast-like cells

**Figure 8:**
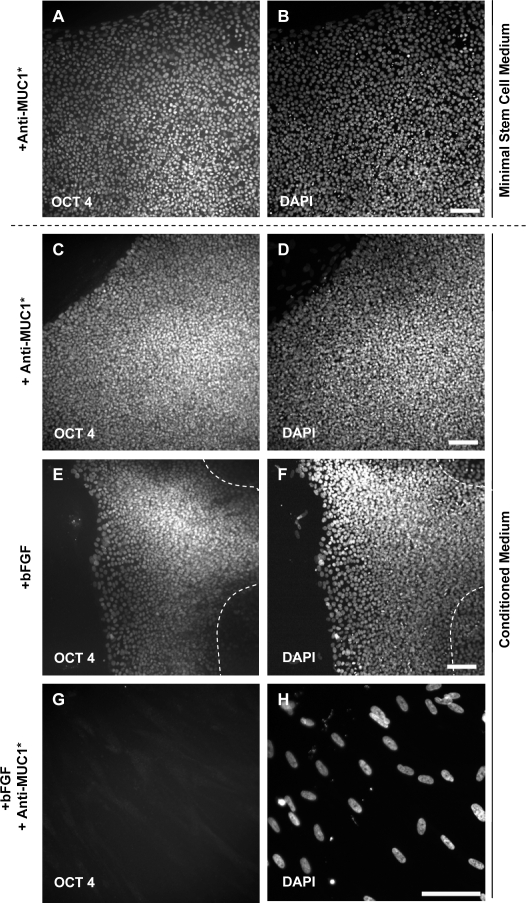
Minimal media plus Anti-MUC1* supports the long-term growth of 100% pluripotent hESCs without added bFGF or conditioned media. H9 hESCs were plated at very low density (4×10^4^ cells per chamber slide well) over matrigel-coated wells then grown for 5 weeks under a variety of test conditions. The resulted colonies were stained with antibodies against OCT4 and DAPI to assess pluripotency. Photomicrographs are shown. A,B. The photo shows a single colony that completely filled the well of the chamber slide when hESCs were cultured in minimal stem cell medium to which Anti-MUC1* was added. OCT4 and DAPI staininig showed that 100% of the cells were pluripotent. The addition of bFGF or Anti-MUC1* and bFGF cultured under the same conditions produced no pluripotent cell growth (data not shown). C,D. hESCS grown in media supplemented with Anti-MUC1* and media supplemented with fibroblast (Hs27)-conditioned medium produced partially as well as completely undifferentiated colonies (pictured). E,F. The addition of bFGF and conditioned medium from fibroblasts, which is standard hESC culture medium for feeder-free systems, produced only partially undifferentiated colonies; dotted lines indicate edge of pluripotent portion of the colony. G,H. Cells grown in conditioned media, bFGF and Anti-MUC1* addition resulted only in OCT4-negative, fibroblast-like cells. Scale bars = 100 µm.

In the absence of conditioned media from fibroblast feeder cells, the only condition that supported the growth of pluripotent stem cells was the addition of Anti-MUC1*, alone (Table 1). The condition that included Anti-MUC1* and bFGF did not produce any pluripotent cells, nor did the addition of bFGF alone. The addition of Anti-MUC1* produced the first colony, the largest colony (completely covered the well) and after five weeks of stimulation with Anti-MUC1* it remained 100% positive for OCT4 (Fig. 8A,B). Stem cells grown under these conditions did not spontaneously differentiate. However, the withdrawal of the Anti-MUC1* antibody, after five weeks, did induce the onset of differentiation. None of the other conditions tested produced *any* OCT4-positive cells when grown in the absence of conditioned media from fibroblasts. Stem cells grown in minimal media that had been supplemented with conditioned media from fibroblast feeder cells produced a mixture of undifferentiated and differentiated colonies in response to treatment with Anti-MUC1* (Fig. 8C,D) or bFGF (Fig. 8E,F). However, treatment with Anti-MUC1* produced colonies sooner and produced more undifferentiated cells than treatment with bFGF. Long-term growth supplemented with bFGF and Anti-MUC1, together, did not support the growth of undifferentiated colonies in either media (Fig. 8E,F).

**Figure 9:**
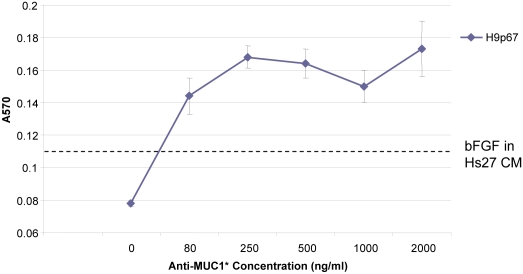
Anti-MUC1* stimulates the growth of pluripotent stem cells in a dose-dependent manner. H9 hESCs at passage 67 were plated at very low density (1.9×10^4^ per well, in triplicate) over matrigel-coated 96-well plates. Cells were cultured in minimal stem cell media, supplemented with Anti-MUC1* antibody to a final concentration of 0, 80 ng/ml, 0.25 ug/ml, 0.5 ug/ml, 1 ug/ml, or 2 ug/ml and cultured for 10 days. Media plus antibody was replaced every other day. After 10 days of growth, cells were stained with Amido Black according to manufacturer's instructions and absorbance at 570 nm was measured on a microplate reader. Stem cell growth was plotted as a function of Anti-MUC1* concentration. The dashed line marks the amount of cell growth that was measured for the control, in which cells were grown in 30% conditioned media from Hs27 feeder cells, supplemented with 4 ng/ml bFGF.

To verify that the stimulation of stem cell growth that we observed was in fact due to the activation of the MUC1* receptor, we measured the stimulatory effect of Anti-MUC1* as a function of antibody concentration. H9 hESCs were plated at 1.9×10^4^ cells/well (in triplicate) on matrigel-coated 96-well plates. Cells were cultured in minimal media without any added fibroblast extracts or growth factors. Anti-MUC1* was added at concentrations that ranged from 0 to 2 ug/ml. Media plus antibody was changed every other day. After ten (10) days most wells had reached 75% confluency. Cell numbers were measured by staining with Amido Black and measuring absorbance at 570 nm. A plot of cell growth as a function of antibody concentration indicates that the bivalent antibody stimulates stem cell growth in a dose-dependent manner (Fig. 9). A control experiment performed in parallel, wherein stem cells were plated at the same density but grown according to standard protocol which included the addition of 30% conditioned media from fibroblast feeder cells and exogenous bFGF. The degree of cell growth that was achieved using the state of the art conditions is denoted on the graph.

Our studies next focused on whether the MUC1* ligand, NM23, could also stimulate embryonic stem cell growth and/or support the growth of pluripotent stem cells in a feeder-free system. H9 hESCs were seeded at 1.2×10^5^ cells/well in 24-well plates and cultured for eight (8) days, supplemented with either nothing, bFGF, Anti-MUC1*, recombinant NM23 or NM23-S120G, which is a mutant that preferentially forms dimers. The results are shown in Table 2. The ligand of MUC1*, NM23, also stimulated and supported embryonic stem cell growth in minimal media devoid of bFGF or feeder-cell extracts. NM23, NM23-S120G and Anti-MUC1* all produced undifferentiated stem cell colonies. Neither the addition of bFGF nor the null control produced any stem cell colonies. As a control, an aliquot of the cells was plated at the same density and grown in 50% conditioned media from Hs27 fibroblasts and bFGF. During the eight-day growth period, this condition generated single undifferentiated cells, but no colonies.

**Table 2:** H9 hESCs cultured in minimal media for 8 days.

Growth Conditions	Confluency	Number of colonies	Morphology
*Minimal Stem Cell Growth Media*			
NM23 1 nM	∼50%	1 colony	1 undifferentiated colonies in 1 of 2 wells
NM23-S120G 1 nM	∼50%	3+2 colonies	3 undifferentiated colonies in 1 well; 2 undifferentiated colonies in the other
anti-MUC1* 80 ng/ml	<50%	1 colony	1 undifferentiated colonies in 1 of 2 wells
bFGF 4 ng/ml	>25%	0	No colonies; fibroblast-like cells
nothing	<25%	0	No colonies; fibroblast-like cells
***Control*** * - 50% Conditioned Media from Hs27 Fibroblast Feeder Cells*			
bFGF 4 ng/ml	<50%	0	No colonies but single undifferentiated cells

Another long-term growth experiment was performed to assess the effects of Anti-MUC1*, NM23_wt_ and NM23-S120G on the rate of stem cell growth and their differentiation state. Results are summarized in Table 3. After four (4) weeks of culturing H14 embryonic stem cells in minimal stem cell media, and in the absence of conditioned media from fibroblast feeder-cells or bFGF, cells treated with either Anti-MUC1* or NM23 grew faster, formed colonies sooner and resisted the onset of differentiation to a far greater degree than state of the art methods.

**Table 3:** H14 hESCs cultured in minimal media for four weeks.

Growth Conditions	Week 1^st^ colony appeared	Number of colonies	Morphology
*Minimal Stem Cell Growth Media*			
NM23 1 nM	Week 2	2 colonies	2 large undifferentiated colonies in 1 of 1 wells; centers of colonies appear to begin to differentiate during week 3; by end of week 4, most of each colony remains undifferentiated
NM23-S120G 1 nM	Week 2	7 colonies	7 large undifferentiated colonies in 1 of 1 wells; centers of colonies appear to begin to differentiate during week 3; by end of week 4, most of each colony remains undifferentiated
anti-MUC1* 80 ng/ml	Week 2	5 colonies	7 large undifferentiated colonies in 1 of 2 wells; centers of colonies appear to begin to differentiate during week 3; by end of week 4, most of each colony remains undifferentiated
bFGF 4 ng/ml	-	0	No colonies
nothing	Week 2	2 colonies	2 very small, differentiated colonies
***Control*** * - 30% Conditioned Media from Hs27 Fibroblast Feeder Cells*			
bFGF 4 ng/ml	Week 2	5	5 mostly differentiated colonies

## Discussion

We have shown that undifferentiated embryonic stem cells do not express full-length MUC1. Rather, they express a low molecular weight cleavage product, MUC1*, which we previously demonstrated has growth factor receptor-like activity on tumor cells. NM23, which was shown to be an activating ligand of MUC1* on cancer cells, co-localizes with MUC1* on pluripotent cells. Unexpectedly, we found that newly differentiated cells no longer express cleaved MUC1* or its ligand, NM23. Newly differentiated stem cells present full-length MUC1. Transition zones between undifferentiated and differentiating cultured stem cells can be found that continue to express OCT4, while also expressing uncleaved, full-length MUC1, which appears to be a marker for the onset of differentiation. Thus, the switch from cleaved MUC1* to the full-length protein may be one of the first detectable signals of the onset of differentiation. These results imply that MUC1* may be a more accurate marker of pluripotency than OCT4 and thus antibodies that recognize MUC1* could be used to search for, identify and isolate pure populations of pluripotent stem cells. Anti-MUC1* has been used extensively in our labs to effectively identify and sort both live and fixed MUC1*-positive cancer cells using FACS. These methods can be readily extended to identifying and sorting live embryonic stem cells, which could automate and improve the procedure for separating out stem cells that remain pluripotent from those that have begun to differentiate. At present this is an imprecise and labor-intensive process that depends on the technician's ability to visually discriminate between cell types then manually dissect pluripotent cells without contaminating the pool with cells that have already entered the differentiation process.

As on cancer cells, MUC1* functions as a growth factor receptor on pluripotent embryonic stem cells. Under conditions that included adding conditioned media from fibroblast feeder cells, antibody-induced dimerization of the extracellular domain of MUC1* stimulated the growth of hESCs more than two-fold better than current methods and importantly without requiring the addition of exogenous bFGF. Further, the addition of MUC1* dimerizing ligands, Anti-MUC1* or NM23, enabled the growth of pluripotent stem cells in feeder-cell-free and bFGF-free minimal growth media. In fact, stem cell growth supported by the addition of MUC1* ligands to minimal media resisted spontaneous differentiation and produced more pluripotent cells than any other growth condition that we tested. In contrast, neither minimal stem cell growth media nor media plus bFGF produced *any* undifferentiated stem cells. Stem cells that were cultured in conditioned media from fibroblasts plus bFGF generated a mixture of undifferentiated and differentiated colonies and the colonies were smaller than those produced by MUC1* stimulation. Thus, in addition to mediating the growth of embryonic stem cells, MUC1* may be a modulator of differentiation. The data presented strongly suggest that MUC1* is a critical marker for the identification and isolation of pluripotent embryonic stem cells as well as a key mediator of the growth and differentiation of pluripotent stem cells.

## Methods

### Anti-MUC1* Antibodies

Polyclonal: Rabbits were immunized with a synthetic peptide corresponding to the first forty-five (45) amino acids of the extracellular domain, GTINVHDVETQFNQYKTEAASPYNLTISDVSVSDVPFPFSAQSGA, conjugated at the C-terminus with KLH and affinity purified by column chromatography. Papain digestion, then purification over a protein A column produced monovalent Anti-MUC1*, collected from the flowthrough (QCB). The specificity of Anti-MUC1* was extensively characterized by Western, FACS, immunocytochemistry, co-immunoprecipitation, and nanoparticle experimentation [1].

### ES Cells and Culture

H9 or H14 hESCs (WiCell) were cultured at 37°C and 5% CO_2_ on either mitomycin-C inactivated Hs27 human foreskin fibroblasts (ATCC) in 6 well plates (BD Falcon). hESC culture media consisted of DMEM/F12/GlutaMAX I with 20% Knockout Serum Replacement, 1% non-essential amino acids stock, 0.1 mM β-mercaptoethanol (all from Invitrogen) and 4 ng/ml human basic fibroblastic growth factor (bFGF, Peprotech). Cells were passaged by manual dissection every 5–7 days at a ratio of 1∶3 and medium was changed every 48 hours. In some experiments, hESCs were grown on matrigel (BD Biosciences) with hESC culture media supplemented with 30% Hs27-conditioned medium and 4 ng/ml bFGF. In other experiments in which Anti-MUC1* was added, conditioned media and bFGF were omitted.

### Immunofluorescence of ES Cells

Manually dissected H9 or H14 cells were plated in 8-well chamber slides (Nunc) either pre-seeded with mitomycin-C inactivated Hs27 human foreskin fibroblasts or pre-coated with matrigel. For undifferentiated cells, cells were fixed 5–7 days after plating. For differentiated cells, bFGF was removed from the culture medium 5–7 days after plating and cells were allowed to differentiate for 14 days before fixation. Cells were washed with PBS prior to fixation with 4% paraformaldehyde in 0.1 M cacodylate buffer for 15 minutes at 4°C. Cells were blocked for 1 hour with 1% BSA and 1% donkey or goat serum in PBS. 0.1% NP-40 was used with antibodies against intracellular antigens. Primary antibodies were diluted in block and incubated with cells for 1 hour at 4°C. The following antibodies were used: OCT4 (Santa Cruz, Clones H-134 and C-10, 1∶100 dilution), SSEA4 (Chemicon, Clone MC-813-70, 2.5 µg/ml), Tra 1–60 (Chemicon, #MAB4360, 2.5 µg/ml), Tra 1–81 (Chemicon, #MAB4381, 5 µg/ml), full-length MUC1 (VU4H5, Santa Cruz Biotechnology, 1∶50 dilution; BD Biosciences, Clone HMPV, 1∶500 dilution) and Anti-MUC1* (Minerva, 1∶250 dilution), control Fab (Jackson ImmunoResearch, #315-007-003), MMP14 (Chemicon, #AB8345, 5 µg/ml), TACE (Chemicon, #AB19027, 5 µg/ml) and NM23 (Santa Cruz, Clone NM301, 1∶100 dilution; BD Biosciences, Clone 56, 1∶100 dilution). Cells were washed 3 times in PBS for 5 minutes prior to incubation for 30 minutes with secondary antibodies: AlexaFluor 488 Goat anti-rabbit IgG, AlexaFluor 555 Goat anti-mouse IgG, AlexaFluor 350 Goat anti-rabbit IgG (Invitrogen, 1∶200); Goat anti-mouse IgM-TR (Santa Cruz, 1∶100). Cells were washed 3 times in PBS for 5 minutes prior to coverslip mounting using an anti-fade mounting medium (Biomeda). Nuclei were visualized by DAPI staining (1 µg/ml) for 5 minutes. Immunostained cells were visualized on an Olympus BX-51 epifluorescent microscope.

### ES Cell Short-Term Growth Assay

#### Quantification of MUC1*-mediated growth

H9 or H14 cells were manually dissected and grown on matrigel-coated wells of a 96 well plate (BD Falcon). Culture media consisted of hESC media supplemented with 30% Hs27-conditioned medium and 4 ng/ml bFGF. Medium was changed and antibodies added every other day at a final concentration of 1 µg/ml for bivalent anti-MUC1* and 100 µg/ml for monovalent anti-MUC1*. Experiments were performed in triplicate. 41 hours-post antibody treatment, live and dead cells were quantified with the LIVE/DEAD viability/cytotoxicity kit (Molecular Probes), following manufacturer's instructions. In other experiments, cells were quantified using Amido Black (Sigma-Aldrich, #A8181). Cells were visualized on an Olympus IX70 inverted epifluorescent microscope and images were captured with a digital camera (QCapturePro). Fluorescence was measured using a Victor3V plate reader (Perkin Elmer).

### ES Cell Long-Term Growth Assays

#### OCT4 immunofluorescence of hESC colonies treated with anti-MUC1*

H9 or H14 cells were trypsin-dissociated and seeded in 8-well chamber slides pre-coated with matrigel at 4×10^4^ cells/well (H9) or 8.2×10^4^ cells/well (H14). Media was changed and antibodies added every other day at a final concentration of 1 µg/ml for bivalent anti-MUC1* until discrete colonies were visible.

Culture conditions include ‘minimal stem cell medium’ (hESC media without feeder-conditioned medium) and Hs27-conditioned medium, with and without bFGF supplementation. For each condition, cells were grown in quadruplicate. Cells were washed with PBS and fixed as described. OCT4 immunostaining was conducted as described.

### Karyotype Analysis of ES Cells

Exponentially growing cultures of cells used in experiments described were prepared in T-25 flasks pre-seeded with Hs27 feeder cells. Karyotype analysis was performed by Cell Line Genetics (Madison, WI).

## Supporting Information

Figure S1 Stem cells used were of normal karyotype. Karyotype analysis of H9 cells at A. passage 50 and B. passage 89 show normal diploid karyotypes.
(0.79 MB TIF)

Figure S2 Two antibodies that recognize different glycosylation states of full-length MUC1 detect full-length protein on differentiated H9 stem cells but not on undifferentiated H9s. A. HMPV antibody that recognizes full-length MUC1 in a glycosylation-independent manner, does not stain undifferentiated H9 stem cell colonies. The dashed line indicates the edge of the stem cell colony. B. Dapi staining verifies that cells are present. C. HMPV stains the differentiated portion of an H9 colony, to the left of the solid line, but not the portion to the right that remains undifferentiated. D. Dapi staining shows that cells are present on both sides of the solid line demarking the border between differentiated and undifferentiated. E. VU4H5 antibody that is able to recognize under-glycosylated full-length MUC1 does not stain an undifferentiated H9 stem cell colony. F. Dapi staining verifies that cells are present. G. Control antibody does not stain. H. Dapi staining. Scale bar = 100 µm.
(6.76 MB TIF)

Figure S3 Two antibodies that recognize different glycosylation states of full-length MUC1 detect full-length protein on differentiated H14 stem cells but not on undifferentiated H14s. A. HMPV antibody that is able to bind to fully glycosylated full-length MUC1, does not stain undifferentiated H14 stem cell colonies. The dashed line indicates the edge of the stem cell colony. B. Dapi staining verifies that cells are present. C. HMPV stains the differentiated portion of an H14 colony, to the right of the solid line, but not the portion to the right that remains undifferentiated. D. Dapi staining shows that cells are present on both sides of the solid line demarking the border between differentiated and undifferentiated. E. VU4H5 antibody that is able to recognize under-glycosylated full-length MUC1 does not stain an undifferentiated H14 stem cell colony. F. Dapi staining verifies that cells are present. Scale bar = 100 µm.
(5.27 MB TIF)

Figure S4 H9 hESCs present a 20 kD MUC1 species that is apparently the cleavage product of MUC1-FL. Lysates were prepared from a single cell clone of MUC1*-1110 (45 amino acids of the extracellular domain) transfected HCT-116 cells and H9 hESCs. Equal amounts of the protein were loaded onto a 12% SDS gel. The gel was run according to standard methods and then blotted with rabbit polyclonal Anti-MUC1*. Both cells produced the characteristic 20 kD MUC1* protein band.
(0.81 MB TIF)

Figure S5 Bivalent Anti-MUC1* stimulates the growth of pluripotent H9 and H14 hESCs, while the monovalent Fab of the same antibody killed essentially all of the stem cells. Undifferentiated H9 and H14 stem cells were cultured in matrigel-coated plates in media supplemented with 30% conditioned media from Hs27 fibroblast feeder cells and 4 ng/ml bFGF. Bivalent Anti-MUC1*, the monovalent Fab of Anti-MUC1*, or a control Fab were added to growing cultures. After twenty-five (25) hours, the number of live cells was measured using a Calcein AM assay wherein fluorescence at 535 nm was recorder on a micro plate reader. A. H9 hESCs. B. H14 hESCS.
(0.55 MB TIF)

Figure S6 Controls for ICC images. A-H are images of secondary antibody controls that were performed as a part of the immunocytochemistry experiments as described and pictured in the figures of the article. Scale bar = 100 µm.
(4.93 MB TIF)
