# Supplementary figures and images for: MUC1* Mediates the Growth of Human Pluripotent Stem Cells
Source: PLoS One. 2008 Oct 3;3(10):e3312. doi: 10.1371/journal.pone.0003312 (PMC2553196; doi:10.1371/journal.pone.0003312)

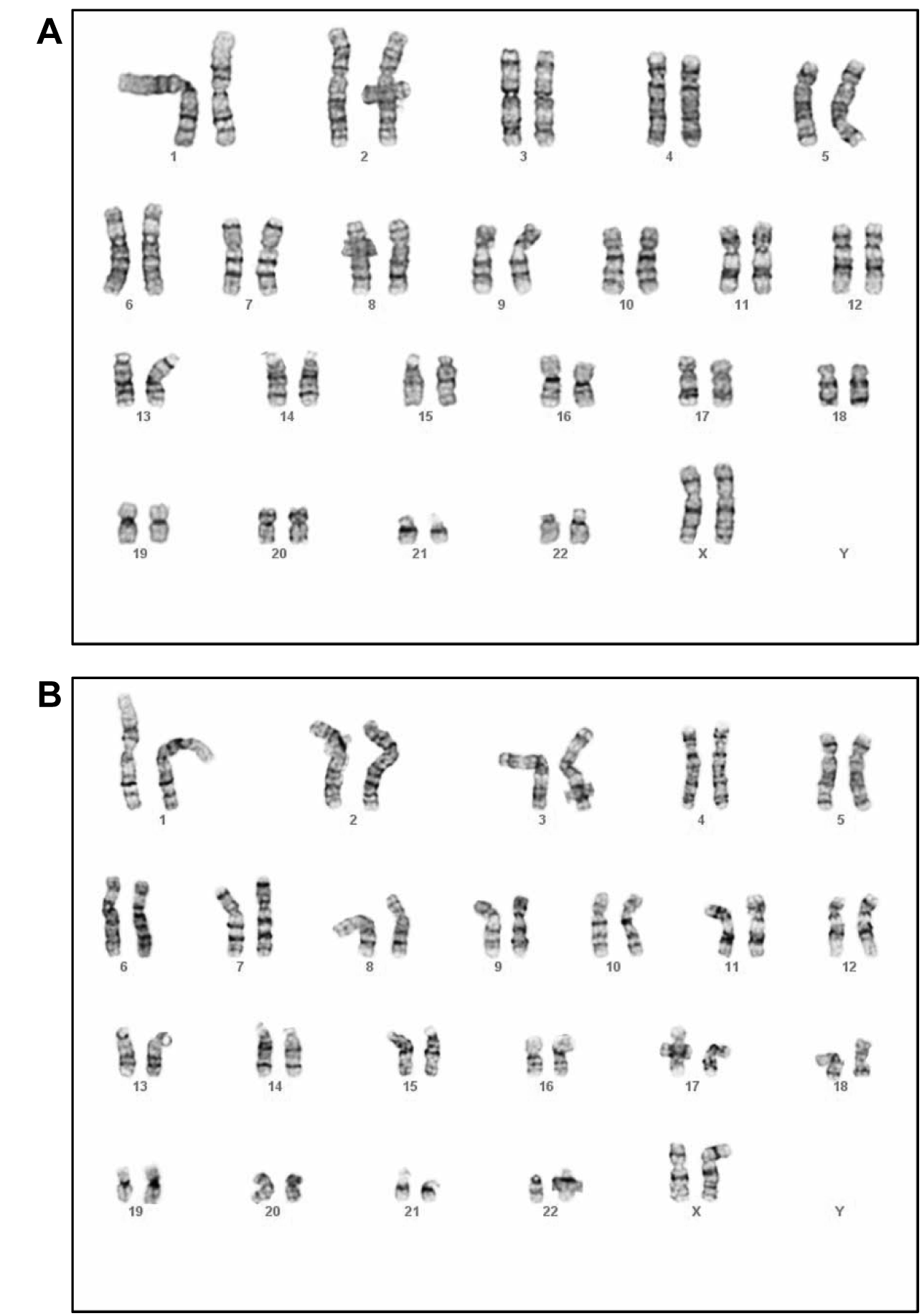

Supplement: Figure S1 — Stem cells used were of normal karyotype. Karyotype analysis of H9 cells at A. passage 50 and B. passage 89 show normal diploid karyotypes. (0.79 MB TIF) [file pone.0003312.s001.tif]

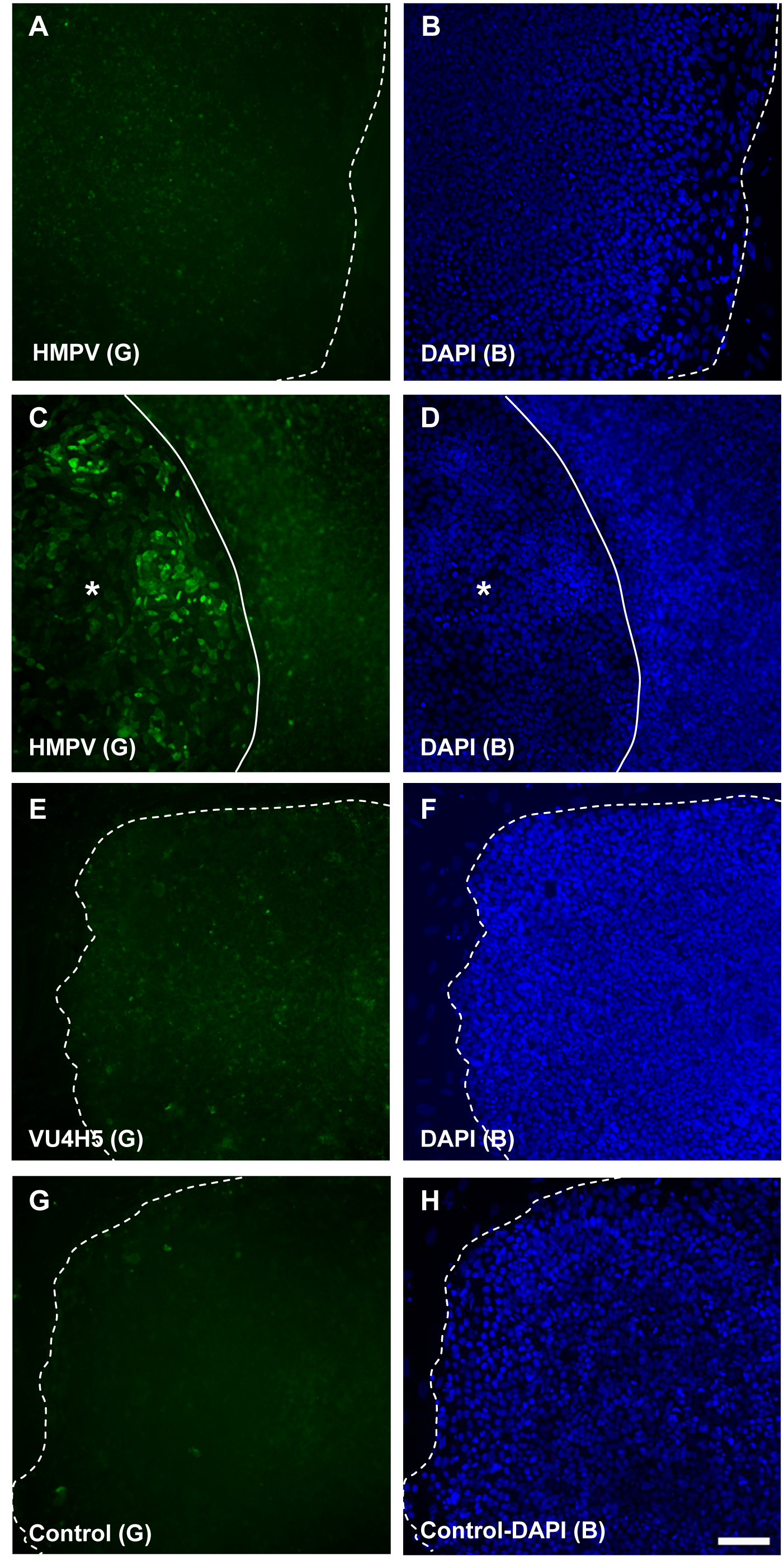

Supplement: Figure S2 — Two antibodies that recognize different glycosylation states of full-length MUC1 detect full-length protein on differentiated H9 stem cells but not on undifferentiated H9s. A. HMPV antibody that recognizes full-length MUC1 in a glycosylation-independent manner, does not stain undifferentiated H9 stem cell colonies. The dashed line indicates the edge of the stem cell colony. B. Dapi staining verifies that cells are present. C. HMPV stains the differentiated portion of an H9 colony, to the left of the solid line, but not the portion to the right that remains undifferentiated. D. Dapi staining shows that cells are present on both sides of the solid line demarking the border between differentiated and undifferentiated. E. VU4H5 antibody that is able to recognize under-glycosylated full-length MUC1 does not stain an undifferentiated H9 stem cell colony. F. Dapi staining verifies that cells are present. G. Control antibody does not stain. H. Dapi staining. Scale bar = 100 µm. (6.76 MB TIF) [file pone.0003312.s002.tif]

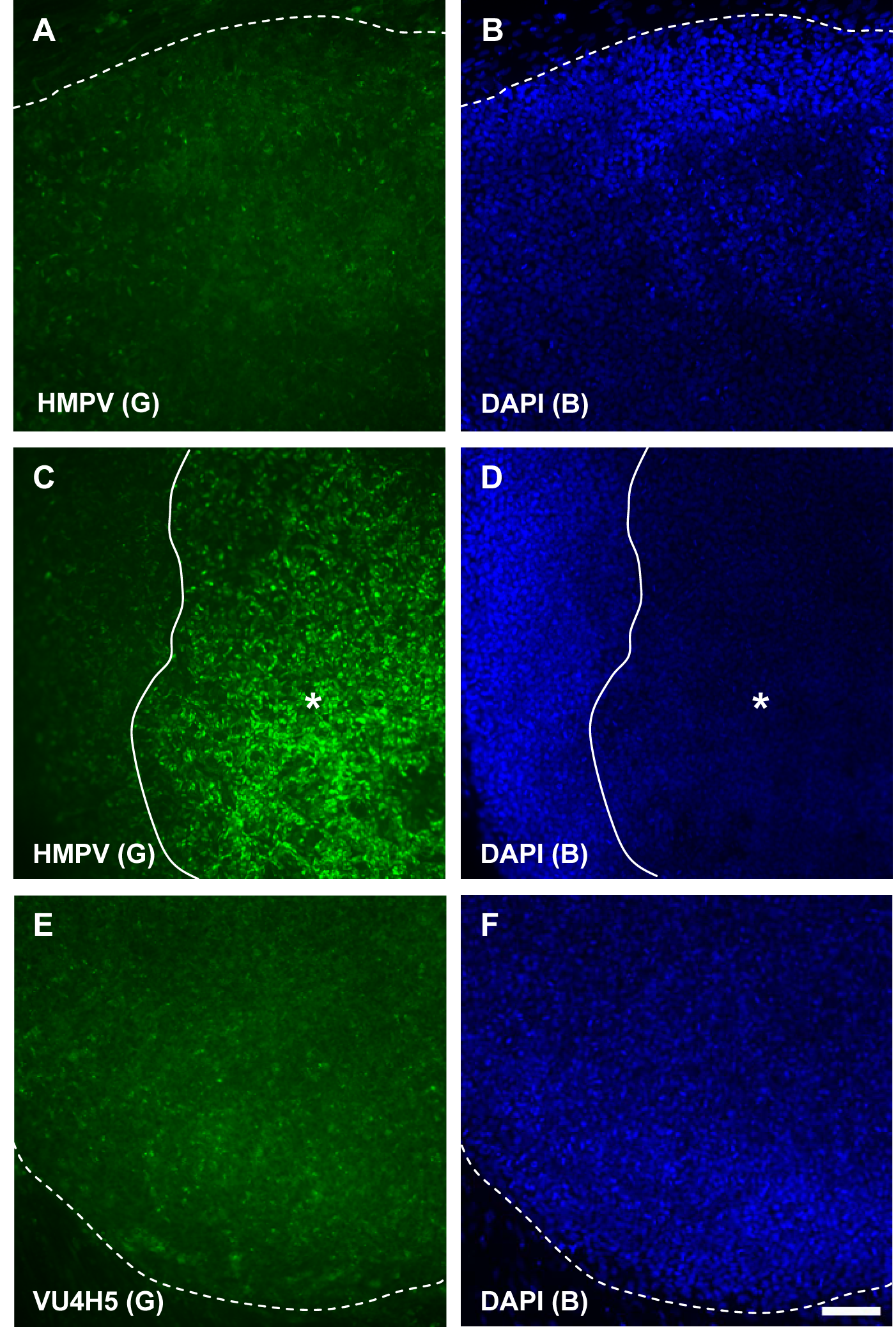

Supplement: Figure S3 — Two antibodies that recognize different glycosylation states of full-length MUC1 detect full-length protein on differentiated H14 stem cells but not on undifferentiated H14s. A. HMPV antibody that is able to bind to fully glycosylated full-length MUC1, does not stain undifferentiated H14 stem cell colonies. The dashed line indicates the edge of the stem cell colony. B. Dapi staining verifies that cells are present. C. HMPV stains the differentiated portion of an H14 colony, to the right of the solid line, but not the portion to the right that remains undifferentiated. D. Dapi staining shows that cells are present on both sides of the solid line demarking the border between differentiated and undifferentiated. E. VU4H5 antibody that is able to recognize under-glycosylated full-length MUC1 does not stain an undifferentiated H14 stem cell colony. F. Dapi staining verifies that cells are present. Scale bar = 100 µm. (5.27 MB TIF) [file pone.0003312.s003.tif]

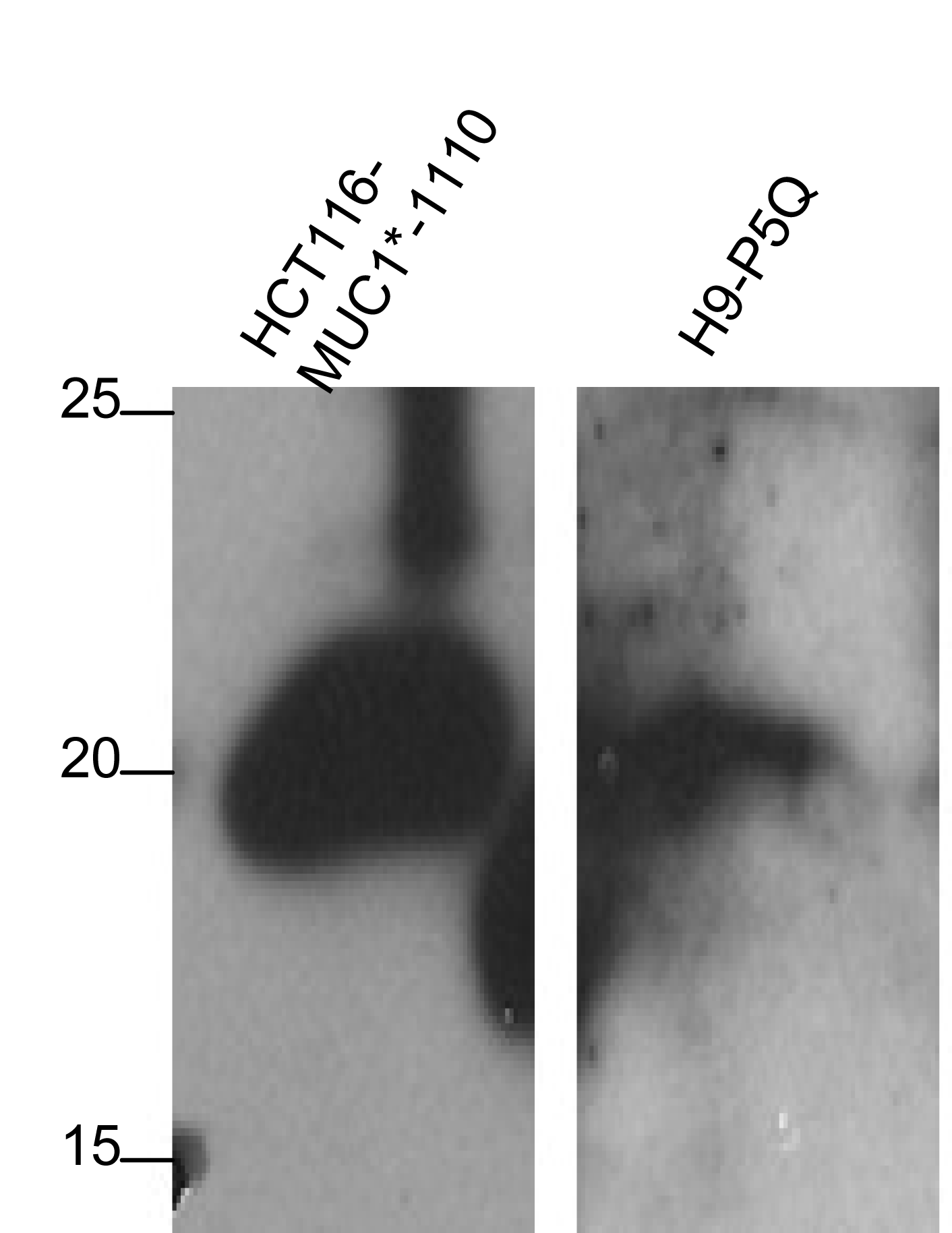

Supplement: Figure S4 — H9 hESCs present a 20 kD MUC1 species that is apparently the cleavage product of MUC1-FL. Lysates were prepared from a single cell clone of MUC1*-1110 (45 amino acids of the extracellular domain) transfected HCT-116 cells and H9 hESCs. Equal amounts of the protein were loaded onto a 12% SDS gel. The gel was run according to standard methods and then blotted with rabbit polyclonal Anti-MUC1*. Both cells produced the characteristic 20 kD MUC1* protein band. (0.81 MB TIF) [file pone.0003312.s004.tif]

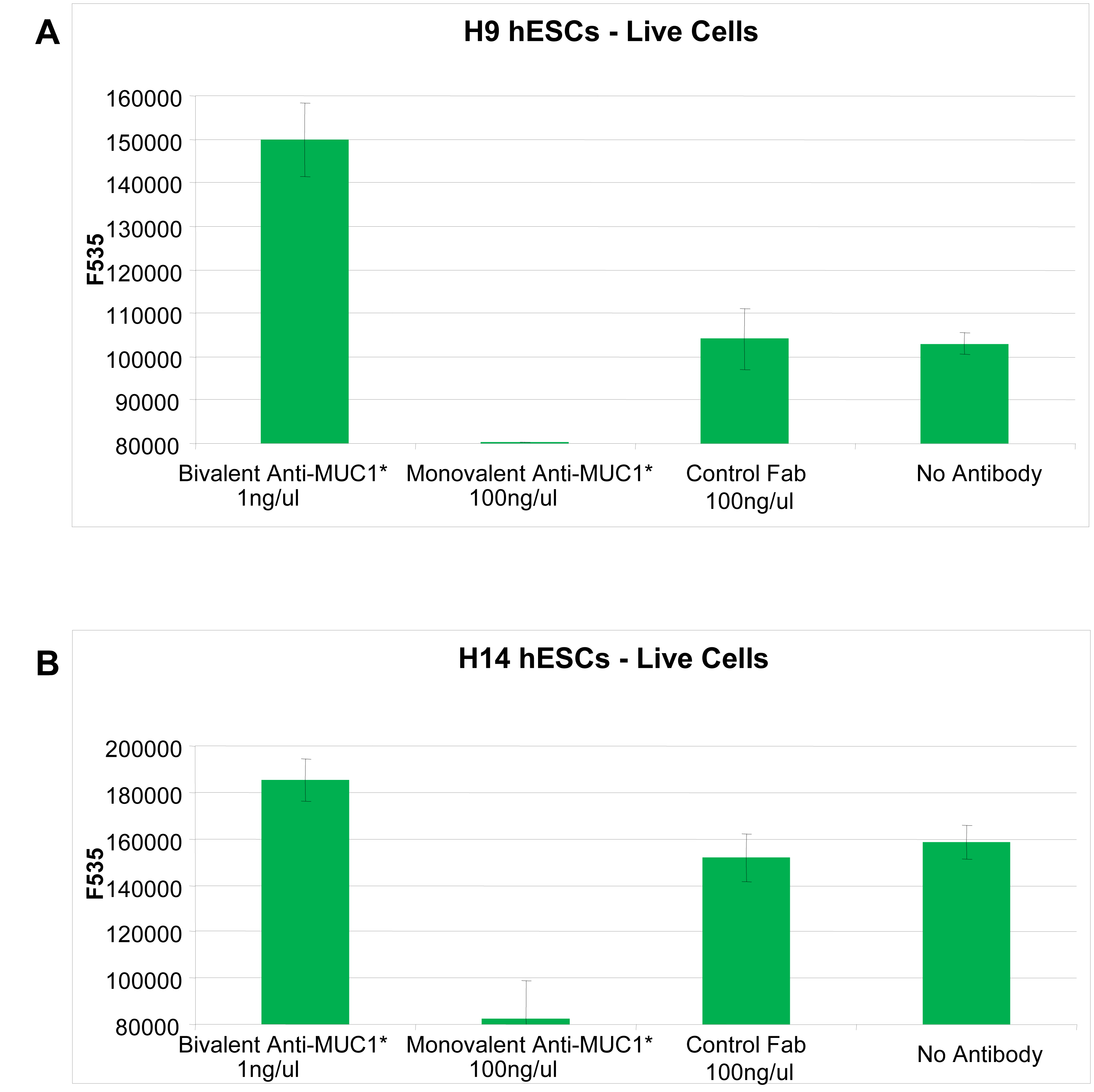

Supplement: Figure S5 — Bivalent Anti-MUC1* stimulates the growth of pluripotent H9 and H14 hESCs, while the monovalent Fab of the same antibody killed essentially all of the stem cells. Undifferentiated H9 and H14 stem cells were cultured in matrigel-coated plates in media supplemented with 30% conditioned media from Hs27 fibroblast feeder cells and 4 ng/ml bFGF. Bivalent Anti-MUC1*, the monovalent Fab of Anti-MUC1*, or a control Fab were added to growing cultures. After twenty-five (25) hours, the number of live cells was measured using a Calcein AM assay wherein fluorescence at 535 nm was recorder on a micro plate reader. A. H9 hESCs. B. H14 hESCS. (0.55 MB TIF) [file pone.0003312.s005.tif]

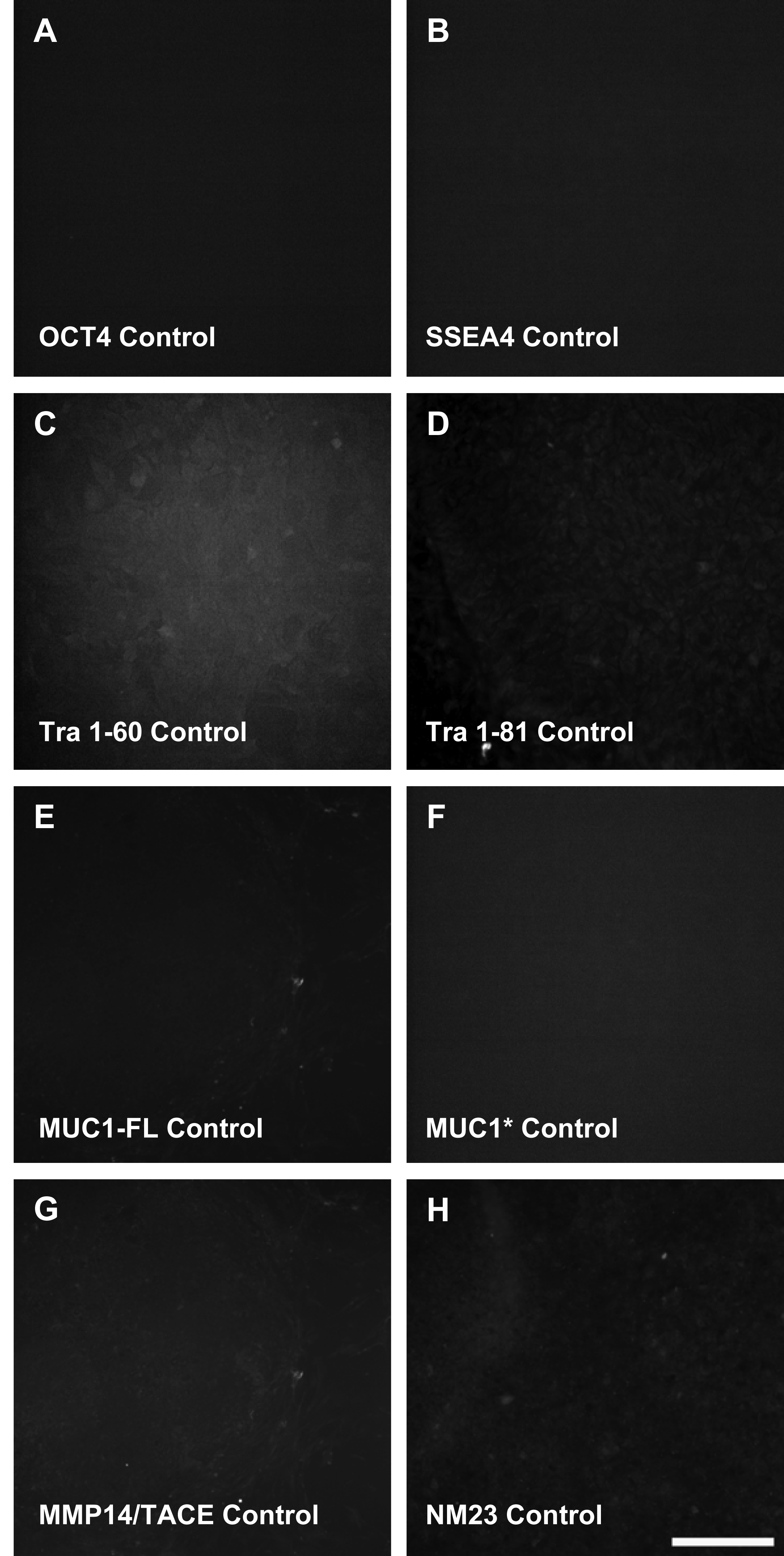

Supplement: Figure S6 — Controls for ICC images. A-H are images of secondary antibody controls that were performed as a part of the immunocytochemistry experiments as described and pictured in the figures of the article. Scale bar = 100 µm. (4.93 MB TIF) [file pone.0003312.s006.tif]
